# Morphological and genetic characterization of jackfruit (*Artocarpus heterophyllus*) in the Kayunga and Luwero districts of Uganda

**DOI:** 10.1186/s12870-024-05064-x

**Published:** 2024-05-09

**Authors:** Racheal Gwokyalya, Ann Nanteza, Henry Wagaba, Siraj Ismail Kayondo, Dan Kazigaba, Grace Nakabonge

**Affiliations:** 1 College of Veterinary Medicine, Animal Resources and Biosecurity, Makerere University, P.O. Box 7062, Kampala, Uganda; 2 National Crops Resources Research Institute, National Agricultural Research Organization, P.O. Box 7084, Kampala, Uganda; 3 International Institute of Tropical Agriculture, Eastern African Hub, P.O. Box 34441, Dar es Salaam, Tanzania; 4 National Forestry Resources Research Institute, National Agricultural Research Organization, P.O. Box 1752, Kampala, Uganda; 5 College of Agricultural and Environmental Sciences, Makerere University, P. O. Box 7062, Kampala, Uganda

**Keywords:** *Artocarpus heterophyllus*, Morphological markers, Microsatellite markers, Crop improvement, Conservation

## Abstract

**Background:**

Jackfruit (*Artocarpus heterophyllus*) is an economically valuable fruit tree in Uganda. However, the production of jackfruit in Uganda is low. Additionally, because of deforestation, genetic erosion of the resource is predicted before its exploitation for crop improvement and conservation. As a prerequisite for crop improvement and conservation, 100 *A. heterophyllus* tree isolates from the Kayunga and Luwero districts in Uganda were characterized using 16 morphological and 10 microsatellite markers.

**Results:**

The results from the morphological analysis revealed variations in tree height, diameter at breast height (DBH), and crown diameter, with coefficient of variation (CV) values of 20%, 41%, and 33%, respectively. Apart from the pulp taste, variation was also observed in qualitative traits, including tree vigor, trunk surface, branching density, tree growth habit, crown shape, leaf blade shape, fruit shape, fruit surface, flake shape, flake color, flake flavor and pulp consistency/texture. Genotyping revealed that the number of alleles amplified per microsatellite locus ranged from 2 to 5, with an average of 2.90 and a total of 29. The mean observed (*H*_*o*_) and expected (*H*_*e*_) heterozygosity were 0.71 and 0.57, respectively. Analysis of molecular variance (AMOVA) indicated that 81% of the variation occurred within individual trees, 19% among trees within populations and 0% between the two populations. The gene flow (*Nm)* in the two populations was 88.72. The results from the ‘partitioning around medoids’ (PAM), principal coordinate analysis (PCoA) and genetic cluster analysis further revealed no differentiation of the jackfruit populations. The Mantel test revealed a negligible correlation between the morphological and genetic distances.

**Conclusions:**

Both morphological and genetic analyses revealed variation in jackfruit within a single interbreeding population. This diversity can be exploited to establish breeding and conservation strategies to increase the production of jackfruit and hence boost farmers’ incomes. However, selecting germplasm based on morphology alone may be misleading.

## Background

The jackfruit (*A. heterophyllus*) tree belongs to the Artocarpus genus. The genus also comprises breadfruit (*A. altilis*), dugdug (*A. mariannensis*), breadnut (*A. camansi*), champedak (*A. integer*) and many other important species [1, 2]. It is a monoecious evergreen crop that bears fruit throughout the year [2, 3]. The highly nutritious fruit contains proteins, energy, fat, carbohydrates, fiber, moisture, vitamin A, thiamine, potassium, calcium, phosphorus, iron, β carotene, riboflavin, niacin, vitamin C, sodium and magnesium [4–6]. This makes it an ideal food supplement during times of food scarcity, especially in rural communities. In addition, various jackfruit parts have anticancer, anti-HIV, and anti-inflammatory effects, and can treat ailments such as anemia, asthma, ulcers, indigestion, dermatitis, diarrhea, cough, tension and high blood pressure [6–11]. The tree also offers agroforestry functions [1, 12] and is a source of latex for making rubber [9]. It is also a source of good quality timber and fuel [13]. Additionally, jackfruit leaves serve as fodder for livestock, and the seeds provide essential nutrients for poultry [14]. Jackfruit trees produce their first fruit at three to eight years after planting and live for up to 60 or 70 years [6, 15]. Every year, the tree produces 700 fruits weighing between 2 and 50 kg each, making them the largest edible fruits in the world. The productivity of the fruit is estimated at 50–80 tons per hectare (ha) of land [16, 17]. In 1992, in India, one of the world’s leading producers of jackfruit, about 1.4 million tons were cultivated on approximately 102,000 ha [16]. In 2010, Bangladesh, another high producer of jackfruit produced 1.5 million tons of fruit, cultivated under an area of 160,000 ha [16], . In Uganda, there is scarce information about the production levels of jackfruit because this fruit has been given little attention. Nonetheless [18], estimated that 0.3 million metric tons of fruit are produced per year per district in some of the regions with high production in Uganda. In the past, jackfruit was grown as a shade tree in homesteads and plantations, but rarely on a large scale [12, 19]. In 1992, the Commonwealth Science Council (CSC) and the International Centre for Underutilized Crops (ICUC) identified jackfruit as an underutilized but diverse crop whose cultivation and conservation require priority promotion [13]. Apparently, the demand for jackfruit is increasing worldwide [16].

Although the origin of jackfruit is believed to be the West Ghats forest of India, the tree is presently cultivated in the Indian subcontinent, Southeast Asia, the Neotropics, northern Australia, the evergreen forest zone of West Africa and East Africa [8, 13, 17, 20, 21]. Jackfruit was probably introduced into East Africa by Asians in the 1890s [22]. In Uganda, jackfruit is predominantly grown in the eastern, central and western regions, although a few trees are found in gardens of homesteads in other parts of the country [18]. Jackfruit is a hardy drought-resistant tree that adapts to a wide range of agroclimatic conditions and survives in the poorest soils, making it a suitable crop in the face of climate change [22, 23]. In Uganda, jackfruit is not only grown for home consumption, as farmers now make products such as dried chips and wine to add value to the fruit, which has a short shelf life [18]. Despite the benefits of the tree, jackfruit production in Uganda still faces challenges. At present, the production of this fruit cannot meet the increasing market demand for export, industry and domestic consumption [18]. Nonetheless, there is potential to improve production because farmers have the desire to plant the tree [18], although they lack elite germplasm. Additionally, as deforestation rises due to increased demand for fuel, timber and land area for agriculture, the loss of tree genetic resources, including jackfruit, is anticipated [24, 25]. In Uganda, there is no jackfruit core collection, as is the case for India, at the Indian Agricultural Research Institute. Having a core collection is paramount for the effective management and preservation of existing genetic resources and as a source of planting material. Hence, there is a need to create planting materials with farmer-desired and consumer-preferred traits, as well as to sustainably utilize and conserve the existing germplasm. These strategies, however, require prior characterization of jackfruit.

This study combined both morphological and genetic markers (SSRs) to characterize 100 jackfruit trees from two major producing districts (Luwero and Kayunga) of the fruit in Uganda [18, 22]. Traditionally, morphological markers have been used for diversity analysis and crop characterization, even before the discovery of DNA-based markers. This is because they are readily available, inexpensive, require simple equipment to screen and are essential for understanding geographic and ecological distributions, evolution and conservation status [26–28]. Morphological markers have been successfully used to characterize jackfruit in India [4, 5, 12, 29–31] and Bangladesh [13, 17, 20, 25]. Additionally, several studies using genetic markers, including amplified fragment length polymorphism (AFLP), random amplified polymorphic markers (RAPDs), and microsatellites, have indicated diversity in jackfruit from various parts of the world [2, 21, 32–34]. In our study, genetic markers were used in combination with morphological descriptors because they are powerful tools for studying phylogenetic relationships. However, genetic markers are more reliable than morphological markers which exhibit continuous variation and high phenotypic plasticity as a result of environmental influence. This is because genetic markers rely on direct analysis of polymorphisms in the species genome, enabling direct measurement of genetic diversity [35, 36]. Microsatellites/SSRs were chosen because they are highly polymorphic, abundant, codominant and reproducible [35, 37]. In this study, therefore, jackfruit was characterized to generate knowledge for designing effective conservation and breeding programmes.

## Materials and methods

### Study area

This study was conducted in the Kayunga and Luwero districts, which are located in the Lake Victoria basin of Uganda. The basin is found in the central region of Uganda, which is known to produce jackfruit in large quantities [22]. The region has a modified equatorial climate type characterized by rainfall throughout the year but with two major seasons (1 and 2). Season 1, from March–May, has more rain than Season 2, from October–December [38]. The mean annual rainfall and temperature range from 1125 mm to 1250 mm and 27.5 °C to 30 °C, respectively. The region has fertile soils that can sustain most crops, and agriculture is the main occupation in the area. Farming in these two districts mostly occurs through subsistence, and the crops grown include bananas, coffee, sugarcanes, maize, sweet potatoes, beans, cassava, tomatoes, pineapples, vanilla, upland rice, groundnuts and vegetables [39, 40].

### Sample size determination

The sample size should be sufficient in genetic diversity studies to avoid biased estimates of allele frequencies and the escape of some alleles, which may be scored as absent [41]. The sample size was determined using the formula below [42]:

$$N= \frac{{\left(z \text{s}\text{c}\text{o}\text{r}\text{e}\right)}^{2 }\times \text{S}\text{t}\text{a}\text{n}\text{d}\text{a}\text{r}\text{d} \text{d}\text{e}\text{v}\text{i}\text{a}\text{t}\text{i}\text{o}\text{n} \times \left(1-\text{S}\text{t}\text{a}\text{n}\text{d}\text{a}\text{r}\text{d} \text{d}\text{e}\text{v}\text{i}\text{a}\text{t}\text{i}\text{o}\text{n}\right)}{{\left(\text{M}\text{a}\text{r}\text{g}\text{i}\text{n} \text{o}\text{f} \text{e}\text{r}\text{r}\text{o}\text{r}\right)}^{2}}$$

where *N* = the necessary sample size, using a standard deviation of 0.1 at a confidence level of 90% and a margin of error of 5%.

$$\text{T}\text{h}\text{e}\text{r}\text{e}\text{f}\text{o}\text{r}\text{e}, N= \frac{{\left(1.645\right)}^{2}\times 0.1 \times \left(1-0.1\right)}{{0.05}^{2}}=97 \text{s}\text{a}\text{m}\text{p}\text{l}\text{e}\text{s}$$

### Plant materials and morphological data collection

Morphological characterization was performed using 16 morphological traits, which are some of the descriptors for jackfruit [42]. These described the tree, leaf and fruit, and included three quantitative (tree height, DBH, and crown diameter) and 13 qualitative (tree vigor, trunk surface, branching density, tree growth habit, crown shape, leaf blade shape, fruit shape, fruit surface, flake shape, flake color, flake flavor, pulp taste and pulp consistency) traits. A total of 100 jackfruit trees (50 from each district) were randomly selected. A minimum distance of 30 m between accessions was used to avoid selecting closely related trees. To select mature trees, only those reported by the farmers to have been bearing fruits for seven years or more were considered. Additionally, among these, only those that had well-developed fruits that were ready for harvesting were selected for accurate assessment of the fruit parameters. From each farmer, only one tree was considered. To study leaf blade shape, fully expanded vigorous leaves, which were visually examined to ensure that they had no disease or nutritional imbalance, were chosen.

The tree height was measured (in meters (m)) from the ground to the top using a suunto clinometer. The DBH was measured (in centimeters (cm)) using a diameter tape. The crown diameter was measured (in cm) as the mean diameter in two directions (north to south and east to west) using a measuring tape. All the qualitative traits were assessed by observation, apart from the pulp consistency and taste, for which farmers’ perceptions were considered. The pulp consistency and taste were described by interviewing the farmers with reference to the ‘descriptors for jackfruit’ [42]. It was emphasized to the interviewees that the description required was for mature fruits and on the first day of ripening because these two traits change depending on the period after harvest. The tree locations were recorded in decimal degrees using the geographical positioning system (GPS) for geographic distance calculations.

### Sample collection, storage and transportation

Two young tender, healthy, and undamaged leaves were collected from each tree at the time of morphological data collection and preserved on herbarium presses. The samples were subsequently transported to the molecular biology laboratory at the National Crops Resources Research Institute (NaCRRI). In the laboratory, the samples were kept at room temperature on a bench to ensure air circulation for proper drying of the leaves.

### DNA extraction and quantification

For each sample, 0.5 g of leaf tissue was ground in liquid nitrogen using a mortar and pestle. Total genomic DNA was extracted from the ground tissue following a modified cetyltrimethylammonium bromide (CTAB) method [43]. Each DNA sample was dissolved in 50 µl of sterile nuclease-free water, and the concentration and purity were determined using a NanoDrop 2000 C spectrophotometer (Thermo Fisher Scientific, Inc., Pittsburgh, PA, USA).

### Amplification and electrophoresis of microsatellite loci

Ten pairs of microsatellite markers designed for breadfruit (*Artocarpus altilis*) by [2] were used in the genetic characterization of jackfruit accessions (Table 1). Polymerase chain reaction (PCR) for amplification of the microsatellite loci was performed using a Biometra T-Professional thermal cycler (Biometra, Göttingen, Germany). A total reaction volume of 10 µl was used. This constituted 1 µl of 10X DreamTaq Green PCR buffer, 0.2 µl of 10 mM dNTP, 0.5 µl of each of the 10 µM forward and reverse primers, 0.05 µl of 5 U/µl Dream Taq polymerase (Thermo Fisher Scientific, Vilnius, Lithuania), 1 µl of DNA (60 ng/µl), and 6.75 µl of sterile nuclease-free water. The PCR procedure was as follows: pre-denaturation at 95 °C for 5 min; 40 cycles of denaturation at 94 °C for 30 s; annealing for 90 s at the temperature optimized for each primer pair (Table 1); extension at 72 °C for 1 min; and a final extension at 60 °C for 30 min. For each sample, 4 µl of the PCR amplicon was electrophoresed along with a 50 bp DNA ladder (NBS Biological Limited, Huntingdon, UK). Electrophoresis was done on 8% *w/v* nondenaturing polyacrylamide gels (PAGE) at 80 V for 8 h in 1X TAE buffer. The electrophoresed gels were stained by immersion in ethidium bromide (0.5 µg/ml) for 30 min and thereafter destained in distilled water for 5 min. The separated DNA bands were visualized and photographed under UV light using the Syngene U: Genius 3 gel documentation system (Integrated Scientific Solutions, Inc., San Diego, CA). PyElph software [44] was used to score the photographed PAGE gels by binary coding. For each locus, only clear bands/alleles with molecular weights within the expected size ranges as reported by [2] were considered.

**Table 1:** Primers used for PCR amplification of jackfruit microsatellite loci [2]

Locus	Primer sequence (5’-3’)	EAS (bp)	T_a_/°C
MAA54a	F: AACCTCCAAACACTAGGACAAC R: AGCTACTTCCAAAACGTGACA	211–239	59
MAA105	F: GTTGGGACACTGTGAACTATTC R: AAAAGCTAGTGGATTAGATGCA	265–293	60
MAA122	F: CTGGCCTTCAGTTTTGTCAAC R: CACCAGGCTTCAAGATGAAA	254–312	55
MAA140	F: CCATCCCCCATCTTTCCT R: TCCTCGTTTGCCACAGTG	142–160	55
MAA145	F: CCAACGCATAGCCAAATC R: AAATCCCAAACCCAACGT	275–299	50
MAA156	F: CTGGTGCTTCAGCCTAATG R: TCAGCGTCAAAGATAACTCG	283–307	55
MAA178a	F: GATGGAGACACTTTGAACTAGC R: CACCAGGGTTTAAGATGAAAC	250–258	55
MAA182	F: TACTGGGTCTGAAAAGATGTCT R: CGTTTGCGTTTGGATAAAT	186–216	54
MAA196a	F: GGAATGTGGTAGATGAAACTCC R: GACAAAAAAACAAAGGAAGAC	283–315	57
MAA196b	F: GAATGTGAGAGATAAATCTCC R: GACAAAAAAACAAAGGAAGAC	337–377	46

EAS = expected allele size, T_a =_ annealing temperature

### Data analysis

#### Morphological data analysis

The raw data were entered into Microsoft Office Excel sheets, from which they were exported to R software version 3.5.1 [45] for analysis. To describe the jackfruit variation patterns, means, standard deviations (SDs), and coefficients of variation for the quantitative traits were calculated. Percentages for qualitative traits were also calculated.

Using the R package ‘cluster’ [46], a distance matrix was constructed to assess the dissimilarity among the jackfruit accessions via the Gower algorithm [47]. The algorithm measures the distances of mixed data (both quantitative and qualitative) simultaneously and takes into account the different scales of measurement of the variables. Cluster analysis was then performed by the PAM/ ‘K-medoids’ method [48] using the Gower distance matrix [47]. Before partitioning, the optimal number of clusters (*k*) to be extracted was calculated using the average silhouette width [49], elbow [50] and gap statistic [51] methods.

Correlation analysis of the morphologic and geographic distance matrices was performed to assess the relationship between morphological appearance and geographic location. Geographic distances were attained by converting the trees’ GPS readings from decimal degrees to Universal Transverse Mercator (UTM) coordinates using UTMConvDutch software [52] and subsequently to kilometers in Genetic Analysis In Excel (GenAIEx 6.503) [53]. Correlation analysis was performed using the Mantel test [54] in GenAlEx 6.503 software [53] with 999 permutations.

### Genetic data analysis

The scoring matrix of the alleles generated by PyElph software [44] was used in GenAlEx 6.503 software [53] to calculate genetic diversity parameters including the total number of alleles, allele frequency, number of alleles at each locus (*N*_*a*_), effective number of alleles at each locus (*N*_*e*_), Shannon’s information index (*I*), proportion of *N* samples that are heterozygous at a given locus (observed heterozygosity (*H*_*o*_)), proportion of heterozygotes expected under random mating (expected heterozygosity (*H*_*e*_)), unbiased expected heterozygosity (*uH*_*e*_) and Wright’s fixation index (*F*) [55]. The AMOVA based on *F*_*st*_ values was also computed to determine the genetic differentiation and the rate of gene flow (*Nm*) in the Kayunga and Luwero jackfruit populations. Significance levels for the variation estimates were calculated using 99 permutations.

In addition, a pairwise individual-by-individual genetic distance matrix was generated and used to perform a PCoA to determine the relationship patterns of the jackfruit accessions. The PCoA was complemented by a dendrogram, which was generated using the R packages “poppr version 2.9.3” [56]. This dendrogram was created using the Provesti distance [57] and the unweighted pair group method with arithmetic mean (UPGMA) [58], with 1000 bootstraps.

The Mantel test in GenAlEx 6.503 software [53], with 999 permutations, was used to determine the isolation by distance (IBD)/the relationship between genetic makeup and geographic location by correlating genetic and geographic distances. The same test was used to assess the relationship between morphological traits and genetic makeup by correlating morphological (Gower) and genetic (Provesti) matrices.

## Results

### Morphological variations of jackfruit

For both the Kayunga and Luwero districts, the highest and lowest variations were observed in the DBH and tree height, respectively, as shown by the coefficients of variation. (Table 2). Characterization of qualitative traits revealed various forms of the assessed morphological characters in both districts. Oblong crown shapes, obovate-shaped leaves and irregularly shaped fruits were unique to the Luwero district. Most of the fruits from Kayunga were strongly flavored, while those from Luwero had an intermediate flavor. Only sweet jackfruit was observed in the entire study region. There was no jackfruit with an insipid, acidic, or bitter taste, as described by [42] (Table 3).

**Table 2:** Variation in quantitative morphological traits of jackfruit in the Kayunga and Luwero districts of Uganda

Plant material	Quantitative trait	Region	Mean	SD	Min	Max	CV (%)
Plant/tree	Height (m)	K	12.6	2.5	7.0	20.0	20.1
		L	12.2	2.5	8.0	19.0	20.8
		**T. P**	**12.4**	**2.5**	**7.0**	**20.0**	**20.5**
	DBH (cm)	K	49.1	20.4	9.7	105.1	40.6
		L	42.7	18.4	19.1	92.4	43.1
		**T. P**	**45.9**	**19.3**	**9.7**	**105.1**	**42.2**
	Crown diameter (cm)	K	3.2	1.0	1.0	5.8	32.4
		L	3.1	1.1	1.3	5.9	35.8
		**T. P**	**3.2**	**1.2**	**1.0**	**5.9**	**33.9**

SD = standard deviation, Min = minimum, Max = maximum, CV = coefficient of variation, K = Kayunga, L = Luwero, T. P = total population

**Table 3:** Variation in qualitative morphological traits of jackfruit in the Kayunga and Luwero districts of Uganda

Plant material	Qualitative trait	Region	Categories of the jackfruit trait (%) [42]
Plant/tree	Tree vigor	K	Low (4.0), Medium (28.0), High (68.0)
		L	Low (6.0), Medium (32.0), High (62.0)
		**T. P**	**Low (5.0), Medium (30.0), High (65.0)**
	Trunk surface	K	Smooth (22.0), Rough (66.0), Very rough (12.0)
		L	Smooth (48.0), Rough (46.0), Very rough (6.0)
		**T. P**	**Smooth (35.0), Rough (56.0), Very rough (9.0)**
	Branching density	K	Sparse (12.0), Medium (56.0), Dense (33.0)
		L	Sparse (26.0), Medium (44.0), Dense (30.0)
		**T. P**	**Sparse (19.0), Medium (50.0), Dense (31.0)**
	Tree growth habit	K	Erect (36.0), semi-erect (12.0), spreading (52.0)
		L	Erect (37.0), semi-erect (12.0), spreading (51.0)
		**T. P**	**Erect (37.0), semi-erect (12.0), spreading (51.0)**
	Crown shape	K	Irregular (34.0), Pyramidal (36.0), Broadly pyramidal (16.0), Semi-circular (8.0), Spherical (4.0), Oblong (2), Elliptical (0.0)
		L	Irregular (44.0), Pyramidal (26.0), Broadly pyramidal (26.0), Semi-circular (2.0), Spherical (2.0), Oblong (0), Elliptical (0.0)
		**T. P**	**Irregular (39.0), Pyramidal (31.0), Broadly pyramidal (21.0), Semi-circular (5.0), Spherical (3.0), Oblong (1.0), Elliptical (0.0)**
Leaf	Leaf blade shape	K	Elliptic (76.0), Narrowly elliptic (16.0), Broadly elliptic (6.0), Oblong (2.0), Obovate (0.0), Lyrate (0.0)
		L	Elliptic (52.0), Narrowly elliptic (36.0), Broadly elliptic (2.0), Oblong (4.0), Obovate (6.0), Lyrate (0.0)
		**T. P**	**Elliptic (64.0), Narrowly elliptic (26.0), Broadly elliptic (4.0), Oblong (3.0), Obovate (3.0), Lyrate (0.0)**
Fruit	Fruit shape	K	Ellipsoid (48.0), Oblong (28.0), Clavate (14.0), Irregular (0.0), Obloid (4.0), Spheroid (6.0)
		L	Ellipsoid (38.0), Oblong (12.0), Clavate (22.0), Irregular (22.0), Obloid (4.0), Spheroid (2.0)
		**T. P**	**Ellipsoid (43.0), Oblong (20.0), Clavate (18.0), Irregular (11.0), Obloid (4.0), Spheroid (4.0)**
	Fruit surface	K	Smooth (6.0), Spiny (94.0)
		L	Smooth (6.0), Spiny (94.0)
		**T. P**	**Smooth (6.0), Spiny (94.0)**
	Flake shape	K	Twisted (36.0), Cordate (18.0), Spheroid (4.0), Rectangular (12.0), Irregular (18.0), Oblong with curved tip (6.0), Obovate (6.0).
		L	Twisted (22.0), Cordate (22.0), Spheroid (22.0), Rectangular (12.0), Irregular (16.0), Oblong with curved tip (4.0), Obovate (2.0).
		**T. P**	**Twisted (29.0), Cordate (20.0), Spheroid (13.0), Rectangular (12.0), Irregular (17.0), Oblong with curved tip (5.0), Obovate (4.0).**
	Flake color	K	Yellow (76.0), Coppery red (16.0), White (8.0)
		L	Yellow (92.0), Coppery red (2.0), White (6.0)
		**T. P**	**Yellow (84.0), Coppery red (9.0), White (7.0)**
	Flake flavor	K	Strong (64.0), Intermediate (22.0), Weak (14.0).
		L	Strong (14.0), Intermediate (46.0), Weak (40.0).
		**T. P**	**Strong (39.0), Intermediate (34.0), Weak (27.0).**
	Pulp consistency	K	Medium (50.0), Soft (42.0), Firm (6.0), Slimy (2.0).
		L	Medium (46.0), Soft (26.0), Firm (22.0), Slimy (6.0).
		**T. P**	**Medium (48.0), Soft (34.0), Firm (14.0), Slimy (4.0).**
	Pulp taste	K	Sweet (100.0), Insipid (0.0), Acidic (0), Bitter (0.0).
		L	Sweet (100.0), Insipid (0.0), Acidic (0), Bitter (0.0).
		**T. P**	**Sweet (100.0), Insipid (0.0), Acidic (0), Bitter (0.0).**

K = Kayunga, L = Luwero, T. P = Total population

### Relationships and clustering pattern of jackfruit accessions

**Fig. 1:**
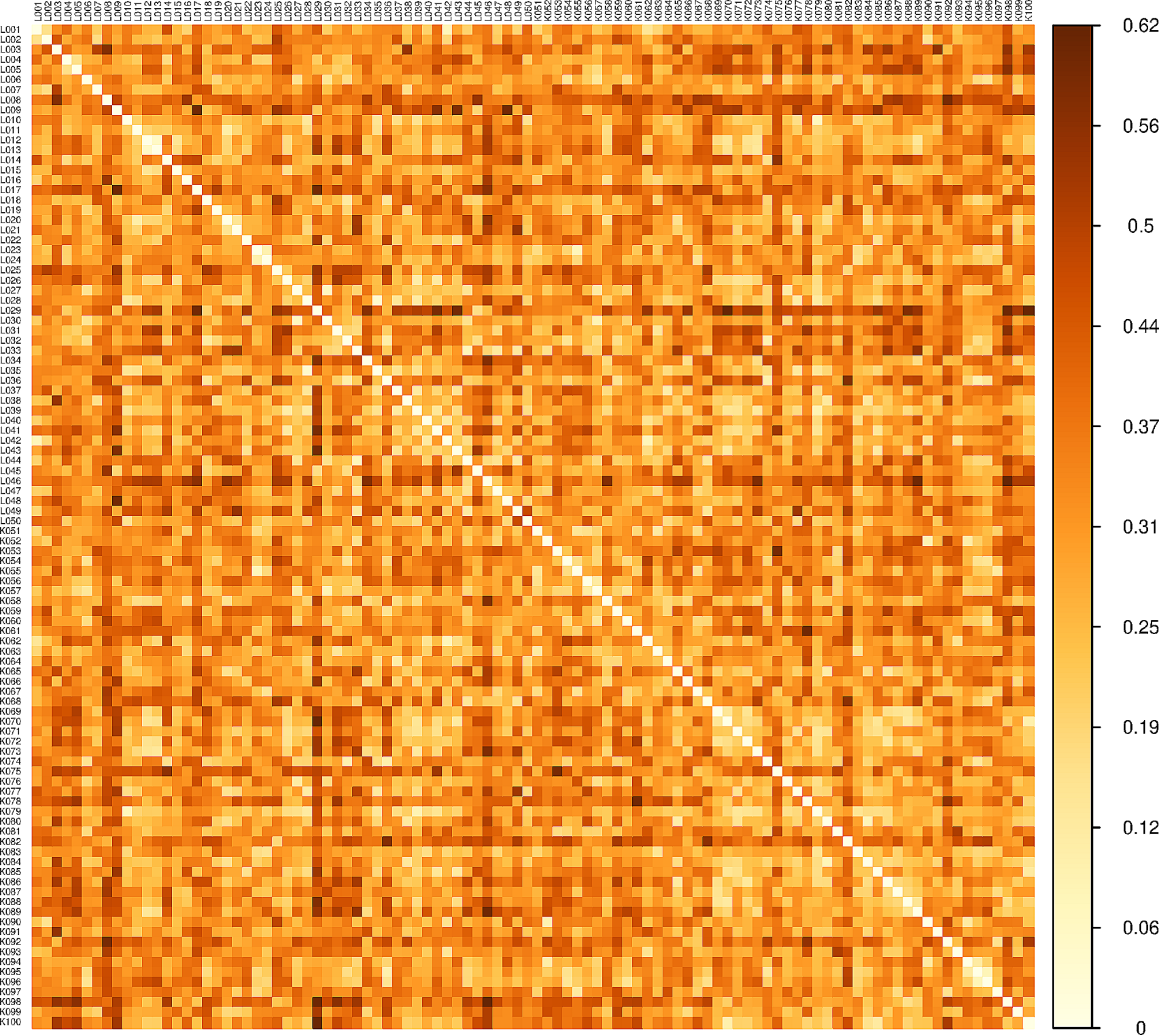
Morphological relationships of jackfruit accessions from the Kayunga and Luwero districts of Uganda. The relationships of 100 jackfruit accessions were derived based on Gower distances. The color intensity is proportional to the distance measure, with the lightest intensity representing the minimum distance and hence close relationships and the deepest intensity representing the maximum distance and therefore distant relationships. The scale on the right side of the plot shows the distance measured with the corresponding colors. The L and K prefixes on the sample identification numbers represent Luwero and Kayunga, respectively

The levels of similarity among the assessed samples varied, as shown by the Gower dissimilarity matrix (Fig. 1). The mean Gower distance of the jackfruit accessions was 0.33. The most similar accessions, which were both from Luwero, had a distance of 0.05 between them, while the most dissimilar (one from Kayunga and the other from Luwero) had a distance of 0.62. Nonetheless, the similarities or variations among the accessions occurred irrespective of their district of origin, as close relationships were observed between jackfruit from different districts and vice versa (Fig. 1). Additionally, the Mantel test revealed that there was no significant linear correlation between the morphological and geographic distances (*R* = 0.034, *p* = 0.170).

**Fig. 2:**
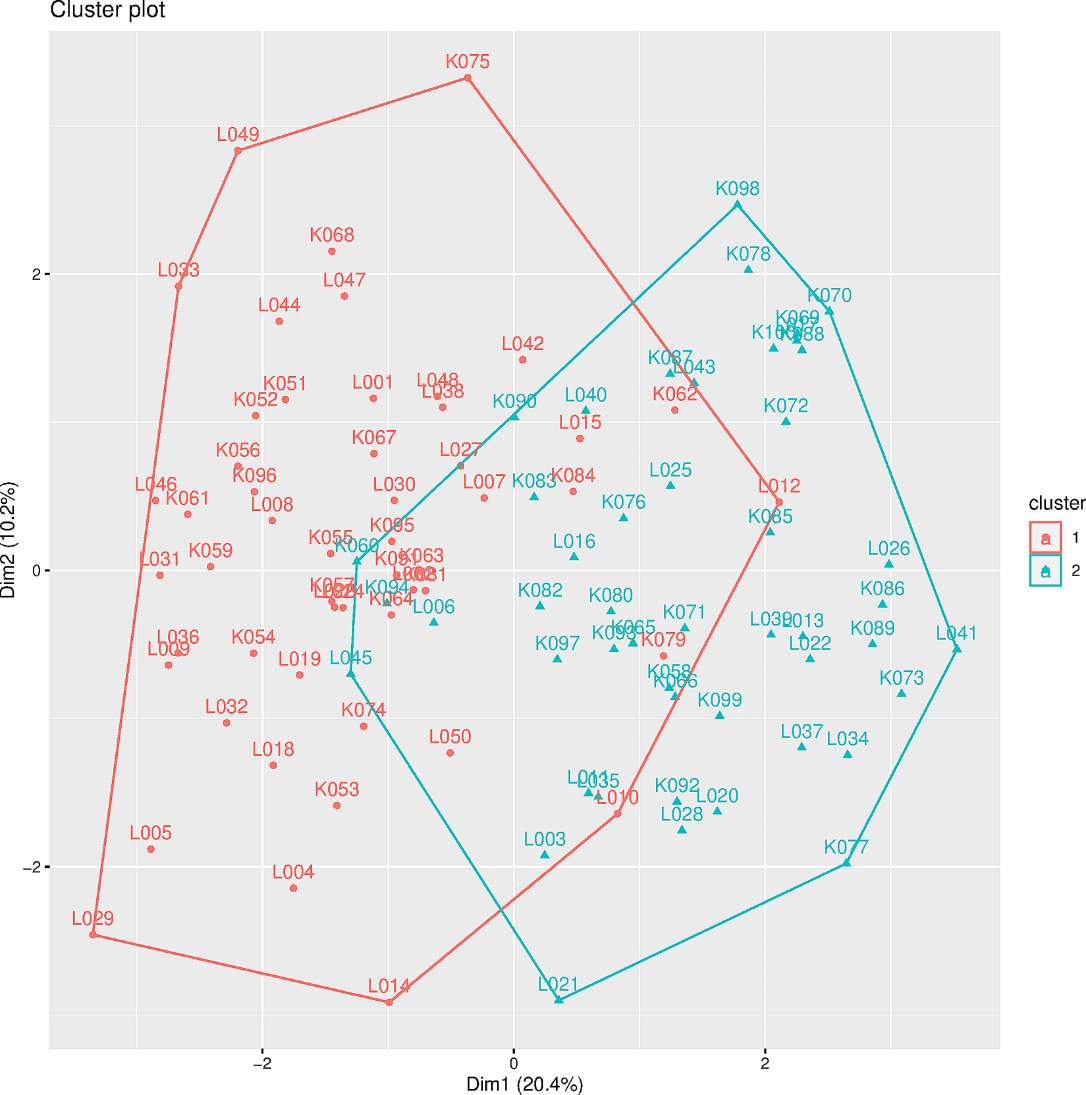
The two clusters of jackfruit accessions from the Kayunga and Luwero districts of Uganda. The clusters were identified via the PAM method [48]. The L and K prefixes on the sample identification numbers represent Luwero and Kayunga, respectively

The three methods used for determining the optimal number of clusters (*k*) to be generated by the PAM [48] were proposed as *k* = 2. The PAM clustering revealed that the two clusters were heterogeneous and that each contained accessions from both Kayunga and Luwero (Fig. 2).

### Genetic diversity of jackfruit

**Fig. 3:**
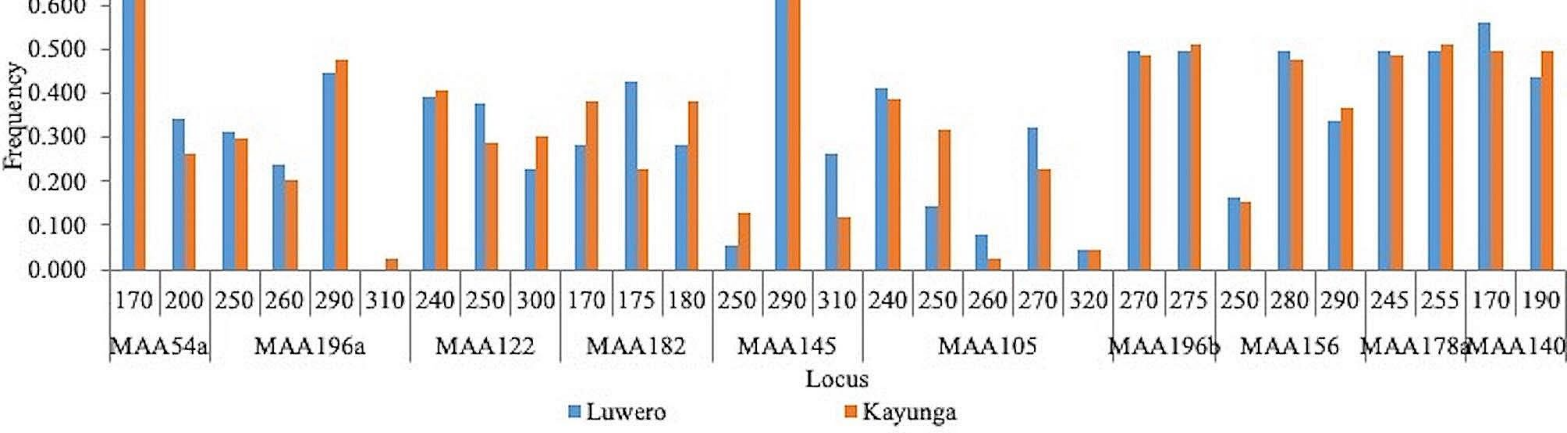
Allele frequency by population across all ten loci. The alleles are coded with numeric names such as “170”, “200”, and “250”. *N* = 100, *n* (Luwero) = 50, *n* (Kayunga) = 50

The assessed genetic diversity indices revealed variation in the jackfruit accessions (Table 4). All 10 microsatellite/SSR markers were polymorphic. A total of 29 alleles were amplified across all loci for 100 samples (Fig. 3). The number of alleles at a locus (*N*_*a*_) ranged from two at loci MAA54a, MAA196b, MAA178a and MAA140 to five at locus MAA105. The mean *N*_*a*_ per locus was 2.90 ± 0.31. The number of effective alleles (*N*_*e*_) ranged from 1.74 at locus MAA54a to 3.42 at locus MAA105, with a mean of 2.42 ± 0.19. The Shannon information index (*I*) was lowest at locus MAA54a (0.62) and highest at locus MAA105 (1.35), and the mean was 0.91 ± 0.08. The least observed heterozygosity (*H*_*o*_) was 0.38 at locus MAA145, while the highest was 0.99 at loci MAA196b and MAA178a. Locus MAA54a had the least expected heterozygosity (*H*_*e*_) and unbiased expected heterozygosity (*uH*_*e*_) (0.43 for both measurements, while locus MAA105 had the highest (0.71 for both measurements. The levels of heterozygosity were higher than expected (the mean *H*_*o*_ was 0.71 ± 0.07, while the mean *H*_*e*_ and *uH*_*e*_ were 0.57 ± 0.03 for both measurements). The mean inbreeding coefficient (*F*_*is*_*)* was − 0.31 ± 0.16. All the loci significantly deviated from Hardy–Weinberg equilibrium (HWE) at *p* < 0.05. Jackfruit variation in Kayunga district did not significantly differ from that in Luwero district, as shown by the mean values of the diversity indices for both districts (Table 4).

**Table 4:** Diversity measurements calculated for the Luwero and Kayunga districts in Uganda

Locus	*N*	*N*_a_	*N*_e_	I	H_o_	H_e_	uH_e_	F	HWE	
									Chisq	*p* value
**MAA54a**	75	2	1.74	0.62	0.61	0.43	0.43	-0.44	14.67	< 0.001
**MAA196a**	80	4	2.81	1.11	0.90	0.64	0.65	-0.40	70.37	< 0.001
**MAA122**	71	3	2.92	1.09	0.51	0.66	0.66	0.23	39.73	< 0.001
**MAA182**	90	3	3.00	1.10	0.68	0.67	0.67	-0.02	180.00	< 0.001
**MAA145**	76	3	1.79	0.78	0.38	0.44	0.44	0.13	81.38	< 0.001
**MAA105**	89	5	3.42	1.35	0.62	0.71	0.71	0.13	116.00	< 0.001
**MAA196b**	69	2	2.00	0.69	0.99	0.50	0.50	-0.97	65.11	< 0.001
**MAA156**	85	3	2.58	1.01	0.52	0.61	0.62	0.15	12.64	= 0.005
**MAA178a**	72	2	2.00	0.69	0.99	0.50	0.50	-0.97	68.11	< 0.001
**MAA140**	80	2	1.99	0.69	0.94	0.50	0.50	-0.88	62.28	< 0.001
**Mean**	78.70	2.90	2.42	0.91	0.71	0.57	0.57	-0.30		
**SE**	2.35	0.31	0.19	0.08	0.07	0.03	0.03	0.16		
**Kayunga**										
**Mean**	40.00	2.90	2.38	0.90	0.71	0.56	0.56	-0.30		
**SE**	1.47	0.31	0.18	0.08	0.08	0.03	0.04	0.17		
**Luwero**										
**Mean**	38.70	2.80	2.41	0.91	0.71	0.57	0.57	-0.31		
**SE**	1.16	0.29	0.17	0.08	0.07	0.03	0.03	0.16		

The least frequent allele (frequency = 0.01), which was also private to Kayunga and rare (with a frequency < 0.05), was “310” of the MAA196a locus (Fig. 3). The most frequent allele was “290” at the MAA145 locus (frequency = 0.72) (Fig. 3). Twenty-eight alleles had frequencies above 0.05 and hence were polymorphic, and no fixed alleles (those with frequency > 0.90) were detected (Fig. 3).

Diversity measurements calculated for 10 microsatellites in 100 jackfruit accessions. *HWE* = Hardy–Weinberg equilibrium, Chisq = chi-square test, SE = standard error.

### Genetic differentiation of jackfruit populations (districts) and cluster analysis

The AMOVA results revealed that 81% of the observed variation was due to heterozygosity within individual accessions. The variation due to differences among accessions within populations/districts was 19%. There was no difference among the Kayunga and Luwero jackfruit populations, as shown by the nonsignificant *Fst* value (*Fst* = 0.003, *P* = 0.19 after 999 random permutations). The average estimate of gene flow (*Nm*) was 88.72.

**Fig. 4:**
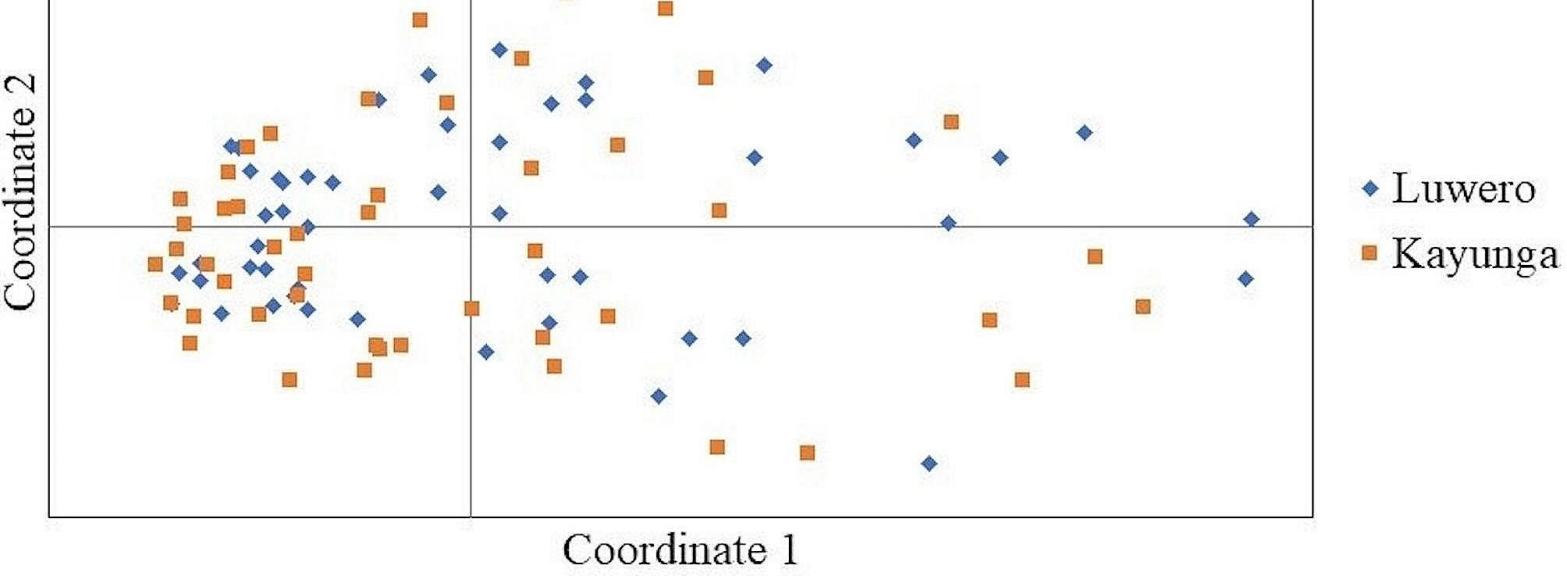
Genetic relationships of jackfruit accessions from the Luwero and Kayunga districts of Uganda. The relationships were determined using 10 microsatellite markers via PCoA. *N* = 100

The lack of distinction between the Kayunga and Luwero jackfruit populations was further shown by the overlapping nature of the accessions from both districts on the PCoA plot. However, the accessions were scattered on the plot, depicting genetic variation (Fig. 4). The combined coordinates explained 38.02% of the total variation, whereby the first coordinate explained 18.28%, the second explained 10.66%, and the third explained 9.09%.

**Fig. 5:**
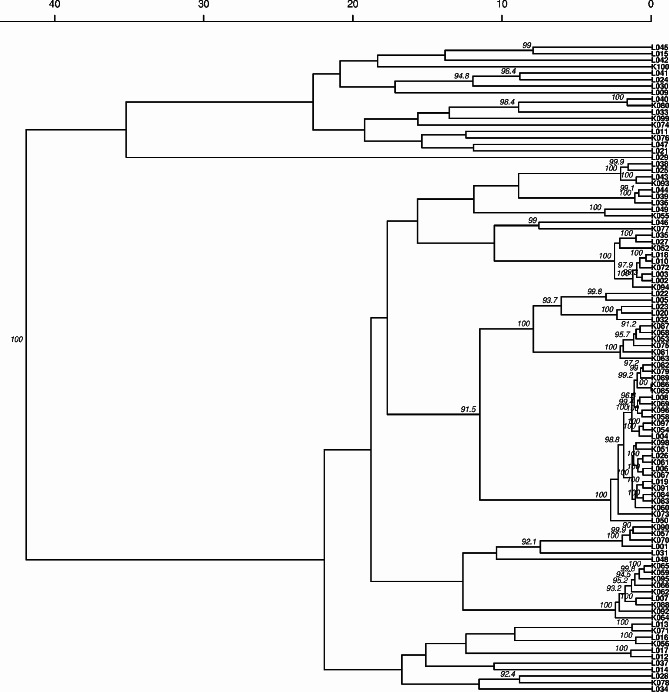
Genetic clustering of jackfruit accessions from the Luwero and Kayunga districts of Uganda. The dendrogram was derived from ten microsatellite loci based on Provesti’s genetic distance [57] and UPGMA [58] with 100 bootstraps. Bootstrap values above 90% are shown. The L and K prefixes on the sample identification numbers represent Luwero and Kayunga, respectively

Like in the PCoA, the dendrogram generated using the genetic distances showed that the clustering of jackfruit occurred irrespective of the district of origin (Fig. 5). In addition, the Mantel test revealed an insignificant correlation between genetic and geographic distances (*R* = 0.035, *p* = 0.240). Additionally, the correlation between the morphological and genetic distances was negligible (*R* = 0.073, *p* = 0.020).

## Discussion

The genetic characterization of jackfruit using molecular markers (microsatellites) was undertaken to generate useful information for breeding programs and for designing sustainable conservation programs.

Diversity in the jackfruit accessions was observed based on the assessed quantitative and qualitative morphological traits. The variation trends of qualitative and quantitative traits in Kayunga and Luwero were similar (Tables 2 and 3). However, the uniqueness of the oblong crown shapes, obovate-shaped leaves and irregularly shaped fruits to Luwero requires further investigation. Similar studies in India [4, 12, 19, 29–31] and Bangladesh [13, 17, 20] revealed morphological variation in jackfruit. More variation in DBH was observed in the present study (CV = 42.2%) than in the study by [31] (CV = 26.90%). Additionally, in the study by [31], most of the trees had medium vigor, smooth trunk surfaces, and ellipsoid tree growth habits, whereas, in the present study, most trees had high vigor, smooth trunk surfaces, and a spreading tree growth habit. No trees with ellipsoid tree growth habits were observed in the present study. The irregular and obloid fruit shapes observed in the present study were not observed in [31]. Spheroid and clavate-shaped fruits were the most common in [31], while ellipsoid fruits were the majority in the present study. In both the present study and [31], spiny fruit surfaces prevailed more than smooth ones. Most of the jackfruit samples studied by [31] had a soft flake consistency, while most flakes in the current study had a medium texture. The differences in jackfruit trees between Uganda and India could be due to the fact that only specific traits (perhaps only those desired) were introduced in East Africa. In addition, as both studies were carried out in specific districts and not countrywide, some traits might have been missed. Jackfruit variation is attributed to cross-pollination and the predominance of seed propagation over a long period [5]. The variation observed is fundamental for germplasm improvement since breeders utilize the existing genetic pool to create crops with desirable traits. For instance, variation in tree height is an important consideration when selecting superior trees because controlling pests and diseases by spraying and harvesting fruits are more difficult for tall trees than for short ones [12]. In this study, trees that were as short as 7.0 m were observed. Additionally, the DBH is an important agronomic attribute to consider when selecting superior trees. The higher the DBH is, the more productive the tree is since the stem supports the branches, which are the fruit-bearing zones. In the present study, trees with DBH values as high as 105.1 cm were observed. In addition, trees with high vigor, which prevailed most (65%) in the present study, survive longer than those with less vigor [13]. In the present study, all categories of the tree growth habit trait, as described by [42], were observed. The tree growth habit is very important when deciding whether a tree is suitable for normal or high-density planting. The latter provides more yield per unit area, and a ‘semierect’ growth habit is suitable for this kind of planting [12]. Additionally, the presence of various flake colors, flavors and pulp consistency categories, as observed in the present study, is important for jackfruit marketability. Most consumers prefer brightly colored and strongly flavored jackfruit, with crunchy or hard pulp, for ready-to-eat snacks [59]. In contrast, soft pulps are preferred for industrial utilization because they require less time and energy to process [18]. There was no variation in the taste of the jackfruit pulp. The fact that the studied trees were domesticated explains why only sweet flakes of jackfruit were observed. The insipid, acidic and bitter jackfruit was probably not domesticated because the taste is not desirable. Nonetheless, the jackfruit trees in Kayunga and Luwero exhibited desirable agronomic traits, which are useful for jackfruit improvement. The observed variation is also important when deliberating on the creation of a core collection and other conservation strategies.

The morphological distance between the two jackfruit accessions, which varied from 0.05 to 0.62 (M = 0.33) (Fig. 1), further confirmed the diversity of the jackfruit accessions. The clustering of the jackfruit accessions was independent of the district of origin (Fig. 2), and closely related jackfruit accessions from either district and distantly related accessions from the same district were observed (Fig. 1). In addition, the correlation between the morphological and geographic distances was nonsignificant (*R* = 0.034, *p* = 0.170). This means that the geographic location of the jackfruit did not influence its morphological appearance. Therefore, factors other than geographic distance, such as cross-pollination, natural selection, and genetic drift, were responsible for the differences in the morphology of the jackfruit, as was observed in similar studies [5, 30]. In addition, the overlapping clusters (Fig. 2) suggested that there was no clear partitioning of accessions into distinct groups; hence, the jackfruit in the two districts belonged to one interbreeding population. Since the two districts are geographically close, with no physical boundary separating them, germplasm exchange may have led to the morphologically similar jackfruit species in these locations. Additionally, considering that morphological appearance is influenced by the environment, similar environmental conditions in the Luwero and Kayunga districts [40, 60] could have led to the similar appearance of the jackfruit in the two locations. Morphological similarity was observed in jackfruit from locations with the same environmental conditions in a study performed in India by [32]. Additionally, cluster analysis of jackfruit in areas of different agroclimatic regions, in the West Ghats of India [5] and Bangladesh [20], generated clear partitioning.

Genetic diversity was revealed by the number of alleles detected, that is 29 alleles with a mean of 2.90 ± 0.31 per locus across the 10 loci for 100 samples. Therefore, as expected for populations with diverse alleles, jackfruit trees from Kayunga and Luwero have long-term potential to adapt and persist in changing environments [61]. However, the study that developed the microsatellite primers used in the present study [2], observed more alleles (116 alleles, with a mean of 8.9 per locus) in an analysis in which the accessions were collected from Thailand, Indonesia, Malaysia, Jamaica, Singapore, Australia, India, Miami and Bangladesh. The greater number of alleles observed in [2] could be explained by the bigger sample size (426 accessions) and the larger geographic area covered than in the present study. Additionally, the difference in the number of alleles observed between these two studies was possibly due to the difference in the electrophoresis methods used. The Beckman Coulter CEQ 8000 and ABI 3730xl DNA Analyzer platforms used in the study by [2] have more resolving power than the PAGE technique used in the present study. In a study by [22] in Uganda, 109 alleles (mean) were observed when SSRs were used to characterize 197 jackfruit trees from different regions of the country. Like in the study by [2], a larger geographical location was covered, and an electrophoresis technique with more resolving power was used, hence more alleles were observed in their study when compared to the present study.

The Shannon information index (*I*) values of 0.91 ± 0.08 and 0.90 ± 0.08 in the Luwero and Kayunga districts, respectively, revealed the rich and even distribution of jackfruit genotypes in the two districts. In agreement, the mean expected heterozygosity (*H*_*e*_) values of 0.57 ± 0.03 and 0.56 ± 0.03 in Luwero and Kayunga, respectively, showed that the diversity levels were similar in both districts. Additionally, comparable levels of genetic diversity were observed by [2] who reported a mean *H*_*e*_ of 0.59. The genetic diversity of jackfruit is attributed to the cross-pollination nature of the tree.

In our study, the mean observed heterozygosity, *H*_*o*_ (0.71 ± 0.07), was greater than the mean *H*_*e*_ (0.57 ± 0.03), indicating the abundance of heterozygotes. The high heterozygosity could be due to excess out-breeding, as shown by the negative *F*_*is*_ index (-0.31 ± 0.16). All the loci deviated from the Hardy-Weinberg equilibrium (HWE) at *p* < 0.05 (Table 4). Similar results were observed for several of the loci by [2]. This observation can be attributed to the fact that jackfruit is a cultivated crop and hence violates several assumptions for HWE due to forces such as natural and artificial selection, nonrandom mating, and gene flow.

The presence of a private allele (allele ‘310’ of the SSR marker MAA196a) in Kayunga was one feature that distinguished the district from Luwero (Fig. 3). Private alleles are indicative of a population’s distinctiveness [61]. Kayunga is likely unique from the Luwero district in terms of the locus MAA196a if the observation is not a result of missing the allele ‘310’ in the latter district due to an insufficient sample size [41]. Therefore, it is worth investigating the allele and determining whether there are more private alleles using a larger sample size and additional populations. The approach taken should not only validate any genetic differentiation among the studied populations but also establish the linkage of the private alleles to important functions that can be targeted in the improvement of the crop.

According to the AMOVA, there was no difference between the Luwero and Kayunga jackfruit populations (Table 5). This was supported by the overlap of accessions from both districts on the PCoA plot (Fig. 4) and the clustering of accessions from either district on the dendrogram (Fig. 5). Furthermore, the Mantel test revealed no isolation by distance (*R* = 0.035, *p* = 0.240), implying that the genotypes were shared across the two districts. The lack of differentiation between the Kayunga and Luwero populations of jackfruit is due to continuous gene flow between the districts, which was reported to occur at an average rate of 88.72%. Gene flow, in the absence of natural selection and genetic drift, leads to genetic uniformity in populations due to the movement of alleles from one population to another [61]. In this case, the gene flow may be attributed to shared germplasm in two geographically close locations, that have no physical boundaries and unintentional seed dispersal by humans [41, 62]. Similar results have been observed in outcrossing species such as *Annona cherimola* Mill [63]. and *Lycopersicon* species [64].

**Table 5:** Molecular variance of jackfruit from the Kayunga and Luwero districts/populations

Source	Degrees of freedom	Sum of squares	Mean squares	Estimated variance	Variation (%)
Among Populations	1	5.07	5.07	0.01	0
Among Individuals	98	401.37	4.10	0.66	19
Within Individuals	100	278.50	2.79	2.79	81
Total	199	684.94		3.45	100

Finally, the correlation between the morphological and genetic distance matrices was negligible (*R* = 0.073, *p* = 0.020), implying that morphological similarity/differences did not guarantee genetic similarity/differences. An insignificant correlation between the morphological and the molecular (SSR) similarity matrices of Persian walnuts was observed by [65]. This finding can be explained by the fact that morphological traits are influenced by environmental factors other than hereditary factors. Another explanation could be that SSR markers exhibit neutral diversity (along the DNA sequence in regions whose function is unknown) and have little association with adaptive variation (diversity based on known genes within the coding regions of the DNA sequence) unless closely linked to genes that control traits [41, 65].

## Conclusions

The results from this study indicate that jackfruit in Kayunga and Luwero is genetically diverse, although the two districts belong to one interbreeding population, as a result of continuous gene flow in these two areas. The diversity observed is useful for the accurate selection of parental lines for hybridization, which, consequently, improves the chances of obtaining superior genotypes when individuals with greater diversity in genetic backgrounds are crossed. Additionally, for the *in-situ* conservation of crops, characterization is essential for identifying populations for preservation as well as the best possible sites for germplasm collection. The allele “310” of the MAA196a locus, which is rare and private to Kayunga, may represent a unique germplasm that requires urgent conservation interventions. Neither morphological nor genetic cluster analyses discriminated jackfruit according to geographic origin. The study also clearly showed that the morphological differences observed in jackfruit are not based on differences in their genetic makeup. Therefore, caution should be taken when selecting germplasms based only on morphological assessments that could be misleading. The findings from this study will be helpful for setting up breeding and conservation programs, which will subsequently boost jackfruit production.

## Data Availability

The datasets used and/or analyzed during the current study are available from the corresponding author upon reasonable request.

## Abbreviations

*A heterophyllus*: *Artocarpus heterophyllus*
AMOVA: Analysis of molecular variance
APAARI: Asia-Pacific Association of Agricultural Research Institutions
CTAB: Cetyltrimethylammonium bromide method
DNA: Deoxyribonucleic acid
dNTPs: Deoxyribonucleotide triphosphates
GenAIEx: Genetic analysis in Excel
GPS: Geographical Positioning System
HWE: Hardy–Weinberg equilibrium
PAGE: Polyacrylamide gel
PCoA: Principal Coordinate Analysis
PCR: Polymerase Chain Reaction
SSR: Simple Sequence Repeat
UTM: Universal Transverse Mercator
